# Textbook of Clinical Neuropsychiatry and Behavioral Neuroscience: Third Edition, by David P. Moore & Basant K. Puri, Hodder Arnold, London, 2012, ISBN-13 978-1-4441-2134-6 (Hardback)

**DOI:** 10.1155/2014/680658

**Published:** 2014-05-06

**Authors:** Jubin Abutalebi

**Affiliations:** San Raffaele University & San Raffaele Scientific Institute, 20132 Milan, Italy

Readers need to be informed of novel, innovative, and ground-breaking books while being warned of books of low quality and those that may even not relate to their specific area of clinical practice and research. The* Textbook of Clinical Neuropsychiatry and Behavioral Neuroscience* fortunately belongs to the former category. It is a ground-breaking and very comprehensive book that covers a wide array of neuropsychological, neurological, and psychiatric disorders including their clinical description, etiology, differential diagnosis, and available treatment. It is now in its third edition. It was previously published as* Textbook of Clinical Neuropsychiatry *and has been retitled and thoroughly updated and extended to include the fundamentals of neuroscience. The very first edition appeared in 2001, the second in 2008, and this one appeared in 2012; hence, there has been more than ten years between the first and last edition and the substantial changes reflect the significant developments that have occurred in this field of science. Like the previous editions, this edition is an excellent clinical guide not only for the behavioral neurologist but also for scholars from related fields such as neuropsychology, neuroscience, and psychiatry. Likewise, students and residents will find in this textbook a useful guide for deepening their knowledge of an immense variety of psychopathological conditions or brain-related disorders such as neurodegenerative and movement disorders, congenital, and developmental brain disorders, vascular disorders, trauma, hypoxic-ischemic disorders, nutritional/toxic/metabolic disorders, infectious and related disorders, prion diseases, endocrine disorders, immune-related disorders, sleep disorders, brain tumour and hydrocephalus, and psychiatric disorders such as mood disorders, anxiety disorders, personality disorders, and substance use disorders.

The book is subdivided into four distinct parts. Part 1 comprises a brief overview of neuroscience fundamentals, which is a completely new section providing background information that encompasses several fields, from neuroanatomy to immunology. Part 2 is dedicated to the diagnostic assessment of neuropsychiatric disorders while Part 3 is dedicated to the study of signs, symptoms, and syndromes associated to brain diseases. Finally, Part 4 provides an extensive survey of specific and distinct diseases.

Part 3 is the most outstanding section of the entire book, providing more than 250 pages of information about signs and symptoms, thus resulting in an excellent resource for residents and students and a very useful text for university courses.

However, the reader has to keep in mind that the* Textbook of Clinical Neuropsychiatry and Behavioral Neuroscience* is not a quick guide on brain diseases but rather a voluminous reference book. Unfortunately, the book does not include much neuroimaging information. Functional (fMRI and PET) and structural neuroimaging (VBM and DTI) methods have been very successful in the past two decades, not only in widening our knowledge on the basic processes underlying most brain functions but also as a powerful tool for the correct diagnosis of many brain diseases. This is something that the authors eventually might consider for a future edition. As it is now, the book does talk less to neuroscientists and researchers and the target readership should be clinically oriented. Finally, purchasing the book provides also free access to an eBook containing all the text and, in addition, a wealth of supplementary material to be exclusively accessed online. It is noteworthy that the book is downloadable not only on computers but also on smartphones and tablets.

In summary, the* Textbook of Clinical Neuropsychiatry and Behavioral Neuroscience* provides a comprehensive book covering clinical neuropsychiatry for students, residents, clinicians, and practitioners of clinical neuropsychiatry and its related fields.

